# *In-vitro *Transcribed mRNA Delivery Using PLGA/PEI Nanoparticles into Human Monocyte-derived Dendritic Cells

**DOI:** 10.22037/ijpr.2019.1100872

**Published:** 2019

**Authors:** Zarin Sharifnia, Mojgan Bandehpour, Hamed Hamishehkar, Nariman Mosaffa, Bahram Kazemi, Nosratollah Zarghami

**Affiliations:** a *Drug Applied Research Center, Tabriz University of Medical Sciences, Tabriz, Iran. *; b *Department of Medical Biotechnology, Faculty of Advanced Medical Sciences, Tabriz University of Medical Sciences, Tabriz, Iran. *; c *Cellular and Molecular Biology Research Center, Shahid Beheshti University of Medical Sciences, Tehran, Iran.*; d *Department of Biotechnology, School of Advanced Technologies in Medicine, Shahid Beheshti University of Medical Sciences, Tehran, Iran. *; e *Department of Immunology, School of Medicine, Shahid Beheshti University of Medical Sciences, Tehran, Iran.*; f *Department of Clinical Biochemistry and Laboratory Medicine, Faculty of Medicine, Tabriz University of Medical Sciences, Tabriz, Iran.*

**Keywords:** PLGA, PEI, IVT- mRNA, GFP, Dendritic cells, Nanoparticle

## Abstract

Induction of protein synthesis by the external delivery of *in-vitro* transcription-messenger RNA (IVT-mRNA) has been a useful approach in the realm of cell biology, disease treatment, ‎reprogramming of cells, and vaccine design. Therefore, the development of new formulations for ‎protection of mRNA against nucleases is required to maintain its activity *in-vivo*. It was the aim of the present study to ‎investigate the uptake, toxicity, transfection efficiency as well as phenotypic consequences of ‎a nanoparticle (NP) in cell culture. NP consists of poly D, L-lactide-co-glycolide (PLGA) and polyethyleneimine (PEI) ‎for delivery of *in-vitro *transcription-messenger RNA (IVT- mRNA) encoded green fluorescent protein (GFP) in human monocyte-derived ‎dendritic cells (moDCs). Nanoparticles that were synthesized and encapsulated with synthetic GFP mRNA, exhibited size distribution in this formulation, with mean particle sizes ranging between 415 and 615 nm. Zeta potential was positive (above 12-13 mV) and the encapsulation efficiency exceeded 73.5%. Our results demonstrated that PLGA/PEI NPs encapsulation of GFP mRNA had ‎no toxic effect on immature monocyte-derived ‎dendritic cells and was capable of delivering of IVT-mRNA into moDCs and was highly effective. The expression of GFP protein 48 h after transfection was confirmed by flow cytometry, microscopic examination and western blotting assay. This NP can make a way to target moDCs to express a variety of antigens by IVT- mRNA. The ‎present study introduced the PLGA/PEI NP, which provided effective delivery of ‎IVT-mRNA that encodes the GFP protein.

## Introduction

Protein pulsing and transfection with *in-vitro* transcribed messenger RNA (IVT-mRNA) are the current strategies for non-viral antigen loading with no risk of altering the host genome. Direct protein delivery is usually not feasible since the size and instability of protein avoids it to provide an adequate concentration with a therapeutic effect in animals. However, ‎IVT-mRNA has some advantages. IVT-mRNA molecules do not exist in nature as they are either chemically modified and/or sequence-engineered. Besides not carrying a carcinogenic risk, IVT-mRNA has temporary activity due to its natural degradation path and does not require transport across the nuclear membrane since it works in the cytoplasm (1). Recently, protein ‎synthesis by the external delivery of IVT-mRNA, which stabilizes expression of the protein in ‎the desired cells, has been a useful approach in cell biology, disease treatment, ‎reprogramming of cells, gene editing strategies, and vaccination. The use of IVT-mRNA as a vaccine stimulates the ‎synthesis of protein antigens to activate the host immune system, effectively eliminating tumor ‎cells, or preventing infection (2-4). The main factors that prevent the advancement of the use of IVT- mRNAs for therapeutic purposes include their instability, immunogenicity and the lack of sufficiently efficacious delivery systems (5). It is expected that these challenges will be reduced by understanding how to modify their untranslated regions (UTRs), and poly(A) tail and study their effectiveness on the sustainability and efficiency of IVT mRNA translation (1).

mRNA vaccines encapsulated in nanoparticles (NPs) whose target cells are DCs may be the most imminent area of vaccination (6, 7). IVT-mRNA can be loaded into hybrid NPs including lipids, polymers, and peptides in a structure that causes stronger ‎transfection (4). Although a few polymers have been considered for formulating biocompatible and biodegradable NPs, FDA approved poly (D, L-lactide-co-glycolide) (PLGA). PLGA is a non-toxic substance and is easily metabolized by the Krebs cycle (8, 9). PLGA NPs of appropriate size are reported to be effectively taken up by mouse and human DCs, and slowly hydrolyzed to induce prolonged antigen stimulation. Now, they are considered candidate for delivery of vaccine (10, 11). PLGA can be easily formulated into NPs (12, 13); however, in order to reduce its limitation in interactions with negative charge compounds such as DNA/RNA, it is necessary to follow the appropriate strategy. Previous studies have shown that the addition of cationic polymer such as Polyethylenimine (PEI) can be used to improve the efficiency of the encapsulation (14). PEI, one of the most positively charged dense polymers‎, has been considered as a potential non-viral delivery vehicle for oligonucleotides, siRNA and plasmid DNA *in-vitro* and *in-vivo*. PEI eases the endosomal release of polymer-DNA complexes in a way that endosome is burst by the particles buffering capacity (pH ranging from 5.0 to 7.2) and the elevated osmotic pressure after acidification of the endosome; consequently, its internal components are released (10, 15 and 16). It was the aim of the present study to prepare a NP consisting of both PLGA and PEI and evaluate the potential of PLGA/PEI NPs for delivery of IVT-mRNA encoding GFP protein. According to our knowledge, the PEI and PLGA mRNA complex has not been previously reported and it can have advantages over the delivery systems described so far.

## Experimental

*Materials*

RiboMAX™ Large-scale RNA Production Systems T7 Kit (P1300) was obtained from Promega (‎Madison, USA). *HindΙΙI* and *GsuI* restriction enzymes were purchased from MBI Fermentas (Germany). Also RQ1 RNase-Free *DNaseI* was obtained from Promega (‎Madison, USA). PLGA with 50:50 monomer ratio with viscosity of 1.05 DL/g and molecular weight of 106 kDa, PEI‎‎ (Average molecular weight 750,000) and polyvinyl alcohol (PVA; Molecular weight 13–23 ‎kDa) were purchased from Sigma-Aldrich (St Louis, MO, USA). RPMI 1640 media and fetal bovine serum (FBS) was purchased from Gibco (USA), penicillin-streptomycin, L-glutamine obtained from Invitrogen (Carlsbad, CA, ‎USA). Total RNA Purification Kit obtained from Jena bioscience (Jena. Germany), Gel Extraction Kit was purchased from Qiagen (Qiagen NV, Venlo, Netherlands). ‎Recombinant human IL-4 and GM-CSF were purchased from Peprotech (Rocky Hill, NJ, USA). ‎ Other chemicals used were among high quality commercial products and used without ‎purification.‎ 

*Methods*

*Synthesis of mRNA Transcripts*

**Figure 1:**
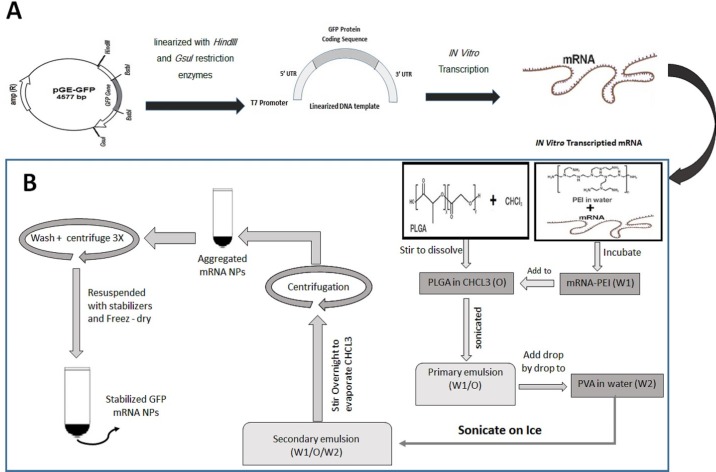
Schematic diagram demonstrating preparation and synthesis of GFP mRNA- encapsulated poly (D, L -lactide- *co *-glycolide) (PLGA) nanoparticles. (A) Preparation of mRNA transcript encoding GFP protein. (B) Synthesis of GFP mRNA- encapsulated PLGA nanoparticles using a double-emulsion solvent evaporation (W1/O/W2) technique

The GFP-encoding plasmid (pGE-GFP) that was prepared in our previous study ‎and consist of ‎‎5’and 3’hsp70 UTRs, T7 promoter, IRES sequences for eIF4G in human dendritic ‎cells and GFP gene, were linearized with *HindIII* and *GsuI* restriction enzymes (17). DNA samples were purified using a PCR purification kit and used as a template for ‎*in-vitro* transcription. mRNA was produced using the RiboMAX™ Large-scale RNA Production ‎Systems T7 Kit. Transcription from 5-10 µg of DNA ‎template was performed in a final 20–200 ‎‎µL reaction mix and incubated at 37 °C for 4 h to generate *in-vitro* transcribed mRNA. mRNA was purified using Total RNA Purification Kit followed by DNase I digestion, ‎according to the manufacturer’s instructions. The quality of mRNA was determined by loading the sample on agarose gel and spectrophotometry. mRNA was stored in RNase-free ‎water at -80 °C. The steps for synthesizing mRNA transcripts are schematically shown in Figure 1A. ‎

*PLGA/PEI NPs synthesis*

The PLGA/PEI NPs with or without synthetic mRNA were formulated using a ‎double emulsion (W/O/W) solvent evaporation method as described previously (18). In this step, mRNA and PEI were mixed and the resultant complex was encapsulated in PLGA nanoparticles. The PEI is added to the ‎PLGA polymer phase (with weight ratio of 30:0.1) to increase the encapsulation rate and improve release ‎of mRNA. Briefly, 4 mg synthetic mRNA mixed with 0.025% PEI in 600 µL final RNase free water reaction and ‎was incubated at room temperature for 30 min. This aqueous solution was mixed with 3% (w/v) PLGA polymer solution ‎and sonicated on ice for the 30 sec to form W/O nanoemulsion. The emulsion from the previous step was added drop wise into the 5 mL of a 2% (w/v) ‎aqueous solution of PVA and the resulted mixture was sonicated on ice for 1 min. This W/O/W emulsion was stirred for at least 12 h at room temperature to evaporate its chloroform. The PLGA NPs were recovered by centrifugation at 20,000 ‎‎× g for 20 min at 4 °C and the supernatant removed and saved for later evaluation. The prepared ‎NPs were washed three times to remove non-encapsulated ‎synthetic mRNA and any residual PVA. The supernatant from each wash step was separately saved to evaluate presence of ‎non-encapsulated RNA in wash solutions. To stabilize the capsulated RNA, the NPs were re-suspended in 5 mL of 5% ‎trehalose solution. At the end, the NPs were sonicated for ‎‎30 sec over an ice bath and the PLGA NPs freeze dried (Alpha 1-2LDplus, Freeze-dryer, Germany). The lyophilized NPs will be stable for 6 ‎months at −20 °C. The steps for synthesizing GFP mRNA PLGA/PEI NPs are schematically shown in Figure 1B. ‎

*Characterization of PLGA/PEI nanoparticle*

*Measurement of size, polydispersity index* *‎* *and zeta potential*

The features of PLGA/PEI NPs including size (diameter), polydispersity index (PDI), and surface charge (or zeta potential) were analyzed using dynamic light scattering, Zetasizer Nano series (Model, Malvern Instruments Ltd., UK). Size of particles was the average of three measurement runs, with triplicate measurements within each run. ‎Moreover, the morphology of the PLGA/PEI particles with and without GFP coding mRNA was examined using a field emission Scanning Electron Microscope (FE-SEM) (s4160, Hitachi‎, Japan). ‎

*Gel retardation analysis*

Gel retardation assay was used to determine complex formation between GFP mRNA and PLGA/PEI NPs (PEI different weight ratio). For this purpose, four microgram of synthetic GFP mRNA and the appropriate amount of PEI were supplemented with RNase free water. The diluted PEI was added to the mRNA solution at different N/P ratios and vortexed. After 30 min of incubation at room temperature, 25 μL of a 3% PLGA solution in chloroform was added to it and 10μL of the complex samples were subjected to electrophoresis on 1% agarose gel containing 0.5 µg/μL ethidium bromide after mixing with loading buffer (30% (v/v) glycerol, 0.25% (w/v) bromophenol blue, and 0.25% (w/v) xylene cyanol FF). Electrophoresis was carried out in 80 V for about 1 h in 1X TBE running buffer. Gel and TBE running buffer were prepared with RNase-Free water. The bands were observed with a transilluminator (Vilber Lourmat E-Box VX2, Marne la Valle´e, France).

*Nuclease protection study*

To determine protection ability of PLGA/PEI for encapsulated GFP mRNA nuclease was added to PLGA/PEI - GFP mRNA complexes to a final concentration 1U nuclease to 4 µg synthetic GFP mRNA and the mixtures were incubated at 37 °C for 1 h. Then SDS solution was added to the samples to a final concentration of 1% to release mRNA from PLGA/PEI-GFP mRNA complexes. Then, the samples were analyzed by electrophoresis on agarose gel 1% containing ethidium bromide and the integrity of the synthetic GFP mRNA in each sample was observed and compared with free synthetic mRNA as control.

*Assessment of entrapment efficiency (EE) and loading capacity (LC)*

The EE of PLGA/PEI- mRNA formulation was assessed by calculating the concentration of ‎free mRNA in the supernatant obtained during the synthesis procedure. To determine the amount ‎of non-encapsulated mRNA (free mRNA), the UV absorbance of all four supernatants, stored from the NPs formulation, was measured EE was ‎calculated using the following Equation: ‎

$\mathrm{EE}\left(\%\right)=\frac{W_{\left(\mathrm{Initial mRNA}\right)}-W_{\left(\mathrm{Free mRNA}\right)}}{W_{\left(\mathrm{Initial mRNA}\right)}}\times 100$

The LC of the NPs was also measured by determining the amount of loaded mRNA in NPs according to this Equation:

$\mathrm{LC}(\%)=\frac{W_{\left(\mathrm{Entrapped mRNA}\right)}}{W_{\left(\mathrm{Total PLGA}/\mathrm{PEI}\right)}}\times 100$

W _(Entrapped mRNA) _is the amount of loaded mRNA in NPs and W _(Total PLGA/PEI)_ is the total amount of PLGA/PEI used in the preparation of NPs.

*In-vitro mRNA release from NPs*

About 1 mg of mRNA-loaded NPs was incubated with 1 mL of release buffer (10 mM Tris–HCl, pH 7.4, 1 mM EDTA) on an orbital shaker at 100 rpm and 37 °C. The samples were taken in triplicate at different predefined time points (*e.g.*, at incubation time, 1, 3, 5, 7, 14, 21 and 30 days). At predetermined intervals, the NPs suspension was centrifuged at 20,000 × g for 20 min at 4 °C. The concentration of mRNA in the supernatant was measured in triplicate for each time point by bio-photometer spectroscopy at the wavelength of 260 nm.

*Generation of monocyte derived dendritic cells (moDCs)*

Human monocyte-derived dendritic cells (MoDCs) were generated as described previously (19). In summary, human PBMCs were isolated from healthy blood donors using Ficoll-Hypaque density gradient centrifugation and were allowed to adhere to plastic plates for 2 h. Adhered monocytes were washed with RPMI 1640 medium and were then cultured for 7 days in RPMI 1640 medium supplemented with 10% ‎FBS, L-glutamine (2 mM), penicillin (100IU), streptomycin (100 µg/mL), recombinant interleukin-4(IL-4:25 ng/mL) and recombinant granulocyte-macrophage colony stimulating factor (GM-CSF: 50 ng/mL). After 7 days, immature monocyte derived dendritic cells expressing iDC-specific markers detected by flow cytometry were ready for NP treatment.

*Nanoparticle uptake assessment*

To test the ability of nanoparticle release from endosomes into the cytosol‎, NPs were labeled with carboxyfluorescein diacetate succinimidyl ester (CFSE) dye. The CFSE is a lipophilic molecule that ‎produces a little fluorescence outside the cell but in cells and in the presence of ‎esterase, it becomes markedly fluorescent (20). Each 1 × 10^6^ moDCs mixed with 100 ng/mL of CFSE labeled PLGA/PEIs NPs and were incubated at 37 °C. Changes in cell-associated fluorescence were examined over time. After 12 and 24 h, 1 × 10^5^ of the cells were analyzed by Flow Cytometer and fluorescence microscopy to confirm NP uptake (Olympus-BX51.USA).

*In-vitro NPs transfection of monocyte derived dendritic cells (moDCs)*

To carry out the transfection experiments, 2 × 10^5^ imDCs was seeded per well in 12-well plate and incubated overnight at 37 °C. Next day, transfection experiments were performed by adding nanoparticles to moDCs. The expression of GFP protein was measured using flow cytometry, fluorescence microscopy (Nikon Eclipse TE2000-U, USA) and western blotting assay 24-48 h after transfection.

*Detection of GFP protein expression in moDCs by Western blotting*

After transfection of immature DCs with IVT GFP mRNA, the transfected and un-transfected cells ‎were degraded in lysis buffer. Centrifuged protein deposit was assessed with Western blot to check the expression of GFP protein. Briefly, the ‎proteins were transferred from SDS- PAGE gel to a nitrocellulose membrane. After incubation of membrane strips in 5% skim milk powder and 0.1% Tween 20 in TBS buffer nitrocellulose membrane incubated with 1:1500 diluted ‎primary antibody (GFP-specific antibody) in 1X TBS. After washing, the strips were incubated for 2 h with‎ 1:5000 diluted secondary antibody (Rabbit polyclonal GFP Antibody- ALP ‎conjugated) in 1X TBS. Finally, the bands were visualized by adding a ‎detection substrate BCIP/NBT solution. Un-transfected cells were analyzed as negative controls ‎for expression of GFP protein. ‎

*Evaluation of PLGA/PEI NPs cytotoxicity based on viability of moDCs*

*In-vitro* cytotoxicity of PLGA/PEI NPs against moDCs at concentrations 5 μg/mL was evaluated using flow cytometry. mDCs cells were stained with the LIVE/DEAD Fixable Far-Red Stain (Near-IR, Invitrogen, UK) based on its fluorescent properties to provide a bright signal when excited with a red laser. The stained cells were analyzed using flow cytometer (FACSAria II, BD, USA) by the appropriate excitation (633/635 nm) and detection channel (780 nm). 

*Statistical Analysis*

Data were analyzed using statistical software (SPSS Version 12; SPSS Software Corporation, Irvine, CA, USA). All data are reported as mean values ± SEM (n = 3). Statistical differences between transfected and un-transfected moDCs were evaluated by ANOVA (Statview; SAS Institute, Cary, NC). Probability values less than 0.05 were interpreted as statistically significant.

## Results

*In-vitro transcription (IVT) process*

**Figure 2:**
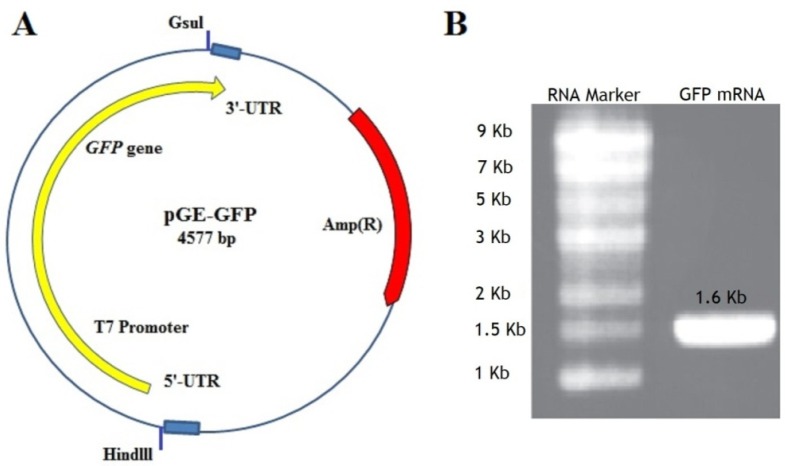
(A) The pGE-GFP plasmid containing *GFP *gene and cis-acting flanking structures such as T7 promoter, the 5 and 3un-translated regions (UTRs) adjacent the ORF. At the beginning and the end of the sequence containing the GFP gene, two restriction enzymes *Hindlll *and *Gsul *have been placed. (B) The GFP mRNA transcript with a length of about 1.6 kb shown on an agarose gel 1.1%. Marker Column: RNA marker with a length of 0.5-9 kb

pGE- GFP containing GFP gene with known flanking sequences (Figure 2A) was digested with ‎*HindIII* and *GsuI *restriction enzymes. The fragments with an approximate length of 1660 bp, were ‎purified using a Gel Extraction Kit and the quality of the purified template DNA was determined by measuring A260/A280 ratio, which was in the range of 1.6 to 1.8. *In-vitro* transcription was ‎performed using the RiboMAX™ Large-scale RNA Production Systems T7Kit using ‎recommended template concentration (5-10 µg). The concentration of synthesized mRNA in a three-time experiment was assayed by spectrophotometric analysis at 260 ‎nm and was between 17.5-‎‎23.5 mg/mL. The quality of mRNA was determined by measuring A260/A280 and visualizing on an agarose gel (Figure 2B). ‎‎

*Nanoparticle characterization*

**Figure 3:**
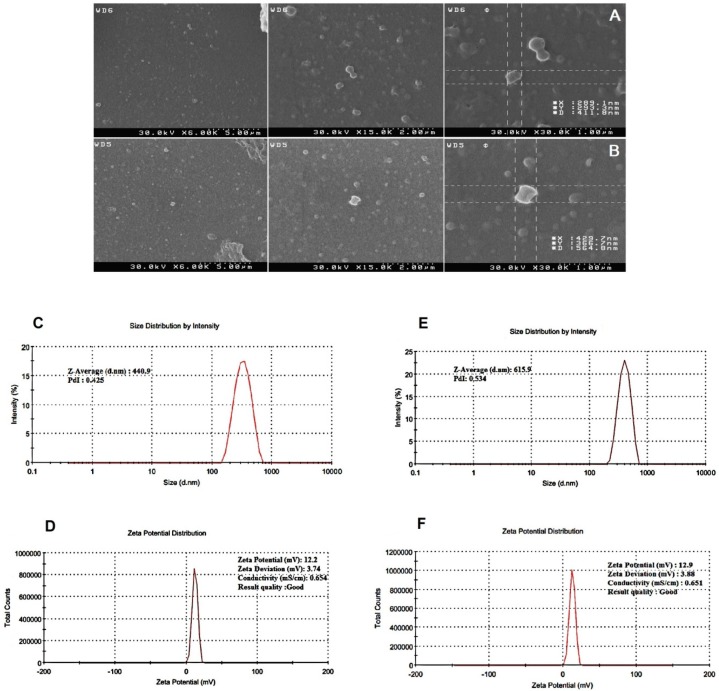
Characterization of PLGA/PEI nanoparticle. Scanning electron microscope (SEM) images showing the spherical morphology of PLGA/PEI nanoparticles (A) Blank PLGA/PEI and (B) mRNA-encapsulate PLGA/PEI. (C) size and (D) zeta potential of Blank PLGA/PEI nanoparticle, (E) size and (F) zeta potential of GFP-encapsulate PLGA/PEI nanoparticle. The images show the values associated with one measurement. Measurements were repeated three times for the formulation of each nanoparticle

**Figure 4:**
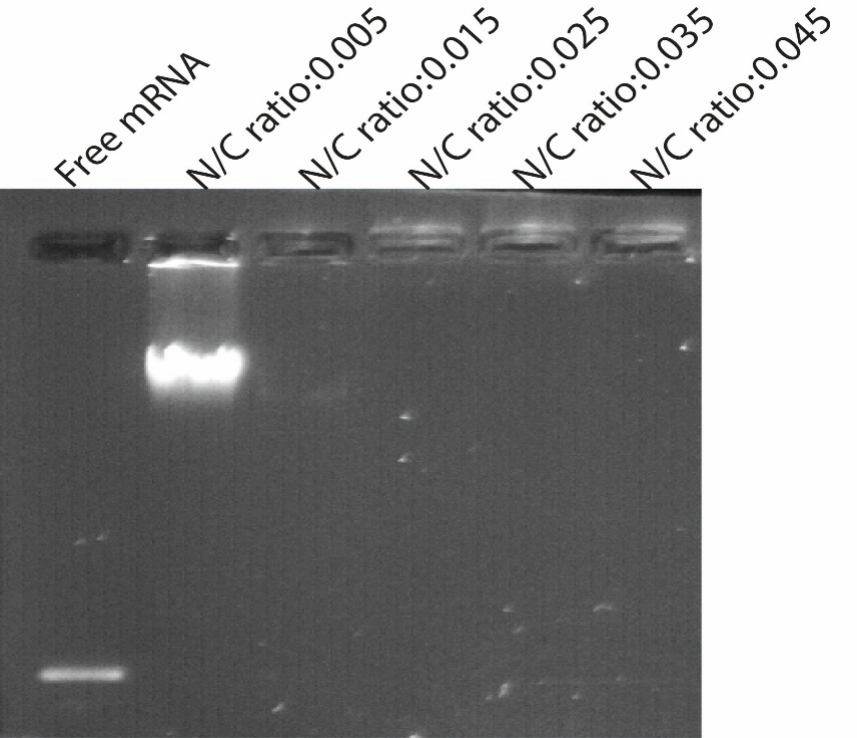
Gel retardation assays. Electrophoretic migration of GFP mRNA complexed with PEI/PLGA at varying N/P ratios ranging from 0.005 to 0.045. Complexes were prepared by mixing 4 μg of synthetic GFP mRNA with different amount of PEI according to the desired ratio. Lane 1 (left) un-complexed synthetic mRNA, lane 2-6 represented GFP mRNA complexed with PEI/PLGA at varying N/P ratios ranging from 0.005 to 0.045. As shown in Figure N/P ratio 0.025 and more than 0.025 were sufficient to totally condense the mRNA, as no free mRNA migrated into the gel. Experiments have been done three times

**Figure 5:**
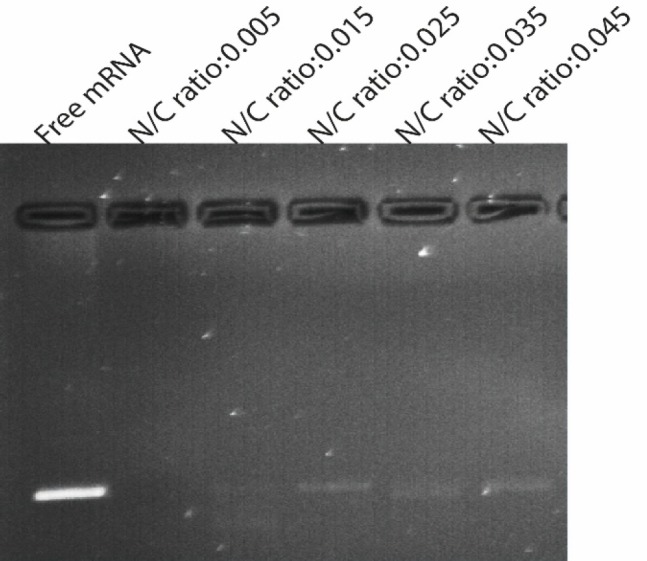
Agarose gel electrophoresis of mRNA extracted from nanoparticles after treatment with nuclease enzyme. Lane 1 (left) untreated control mRNA, lane 2-6 represented GFP mRNA complexed with PEI /PLGA at varying N/P ratios ranging from 0.005 to0.045 incubated with nuclease at 37 for 1 h. As shown, N/P ratio 0.025 and more than 0.025 was sufficient to protect mRNA from digestion by nuclease

SEM images showed that the NPs are spherical ‎and are well dispersed showing homogeneous ‎distribution around 400–600 nm in diameter and that the encapsulation of synthetic mRNA did ‎not seem to cause morphological alterations. Also a small amount of particle agglomeration was observed (Figures 3A and 3B). The results of the determination of size and zeta potential for PLGA/PEI particles with and ‎without mRNA are shown in Table 1. Size and zeta potential were reported as the average ‎and standard deviation of the measurements, with repeated three times per sample. Size of blank ‎PLGA (particle without mRNA) and mRNA-encapsulated PLGA/PEI NPs is in average ‎‎428.9 ± 12 and 606.45 ± 9.7 nm in diameter, respectively. The polydispersity index (PDI) indicated ‎that mRNA-encapsulated PLGA/PEI NPs (PDI of 0.454 ± 0.084) were distributed slightly higher than blank PLGA/PEI NPs ‎‎ (PDI of 0.437 ± 0.012). Measured zeta potential values were measured for both blank PLGA/PEI ‎and mRNA-encapsulate PLGA/PEI to be 12.9 ± 0.275‏‎ mV and 12.2 ± 0372 mV (Figures 3C-3F). Then, in order to better understand the behavior of these nanoparticles in the living environment, the mRNA complex was prepared with different polymer ratios. First, the condensation of the synthetic GFP mRNA by PEI at various different N/P ratios (0.005-0.045) was analyzed by gel retardation assay (Figure 4). It is well established that during electrophoresis, the complexes, which are less negatively charged and heavier than free mRNA, are retained in the wells (N/P ratio 0.025-0.045) whereas un-complexed mRNA can migrate into the gel. As shown in Figure 4, our results demonstrated that an N/P ratio 0.025 was sufficient to totally condense the mRNA, as no free mRNA migrated into the gel. Although the N/P ratio of more than 0.025 was sufficient to condense all of the mRNA, due to the high toxicity of PEI, the best ratio for transfection in the cultured cells was considered to be N/P ratio 0.025. Also, maintaining the mRNA in nanoparticles in the presence of nuclease for 1 h incubation was investigated. As shown in Figure 5, our results indicated that N/P ratio of more than 0.025 was sufficient to protect mRNA from digestion by nuclease. This result demonstrated that PLGA/PEI nanoparticles could protect encapsulated mRNA from nuclease digestion.

*IVT-mRNA EE and LC determination*

The mRNA encapsulation rate in NPs can be calculated by subtracting the amount of recovered mRNA in the wash supernatants from the initial amount of mRNA used. In four supernatants obtained during the NP formulation process, non-encapsulated ‎mRNA remaining was evaluated. Absorbance measurements at 260 nm by UV spectrophotometer were obtained for all four supernatants. The remaining mRNA level was measured in the range of 1140 to 1340 μg in supernatant and the calculated average values of encapsulation efficiencies (EE%) and loading capacity% (LC%) were 73.54 ± 2.12% and 11.47 ± 0.33 (Table 1).

*In-vitro mRNA release study*

**Figure 6:**
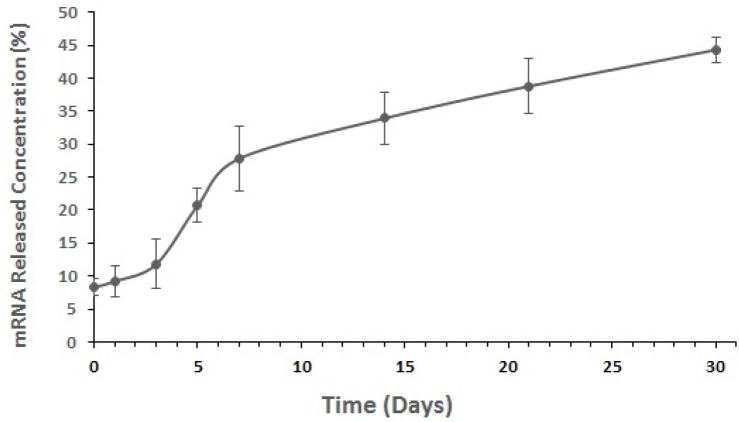
*In-vitro *release curves of mRNA from the optimized nanostructured PLGA/PEI formulation. Measurements were repeated in triplicate for each time

Figure 4 shows that by considering the initial amount of loaded mRNA (3500-‎‎4700 μg) and EE%, the mRNA release rate from ‎nanoparticles during 30 days after encapsulation was about 8.3–44.3% which is considered as a slow process (Figure 6).

*Particle uptake*

**Figure 7:**
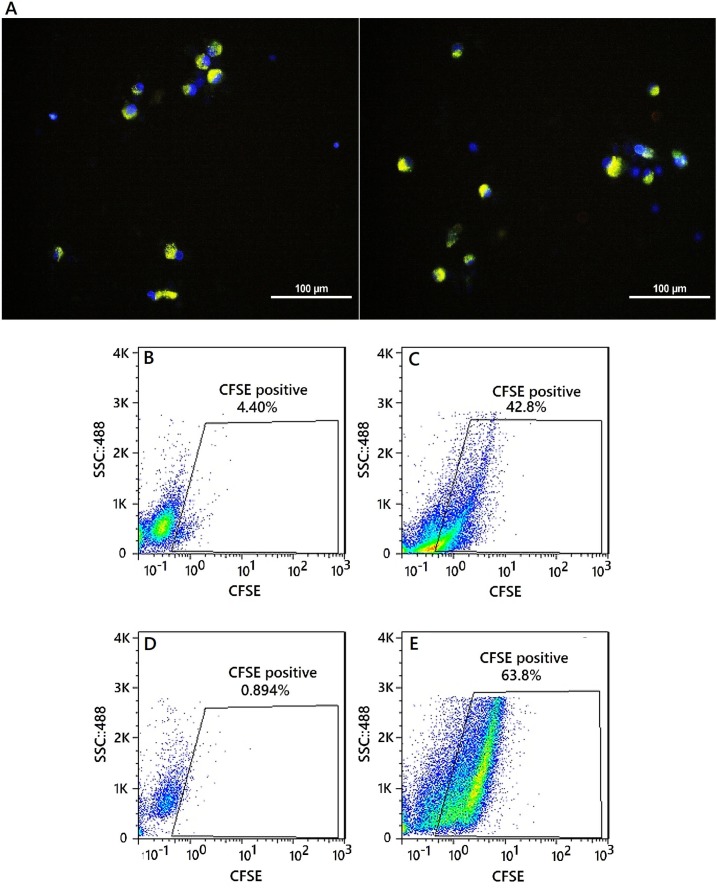
Intracellular uptake of CFSE-encapsulating nanoparticles by moDCs. (A) Fluorescent images of uptake of CFSE-encapsulating nanoparticles by moDCs. Merged DAPI and CFSE image. Nanoparticles are greenish-yellow and the nucleus is blue, nuclei stained with Hoechst 33258. Scale bars represent 100μM. Flow cytometry analysis of moDCs encapsulated the CFSE labeled nanoparticles. (B andD) CFSE-positive moDCs were not exposed to the nanoparticle and gated in a CFSE-(FL1) dot plot (Day 0). (C) CFSE-positive moDCs were exposed to the nanoparticle (12 h after the exposure - percentage of positive cell: 42.8%). (E) CFSE-positive moDCs were exposed to the nanoparticle (24 h after the exposure - percentage of positive cell: 63.8%)

To show the uptake and endosomal escape of NPs in moDCs, the NPs were labeled by CFSE dye. Immature moDCs were incubated with labeled NPs. After 12 h incubation, moDCs were collected and ‎stained with Hoechst dye. The mRNA uptake efficiency of this NP was quantitatively analyzed using a FACS Calibur flow cytometer. Fluorescent intensity of moDCs transfected with labeled NPs compared to the control cells, which were moDCs that did not have exposure to labeled NPs. Using this method, the introduction of NPs into the cytoplasm of the moDC cells was ‎confirmed because the CFSE-labeled nanoparticles show its fluorescence property upon entry ‎into cytosol. The moDCs, recieving labeled NPs were washed ‎‎3 times with PBS and their fluorescence emission intensity was measured after 12 h and 24 h. Analysis by fluorescence microscopy exhibited localized fluorescence signals after ‎culturing moDCs (Figure 7A). Also the percentage ‎of CFSE-positive moDCs 12 and 24 h after the exposure were 42.8%, 63.8%, respectively (Figures 7B-7E).

*Performing of mRNA transfection with NPs*

**Figure 8:**
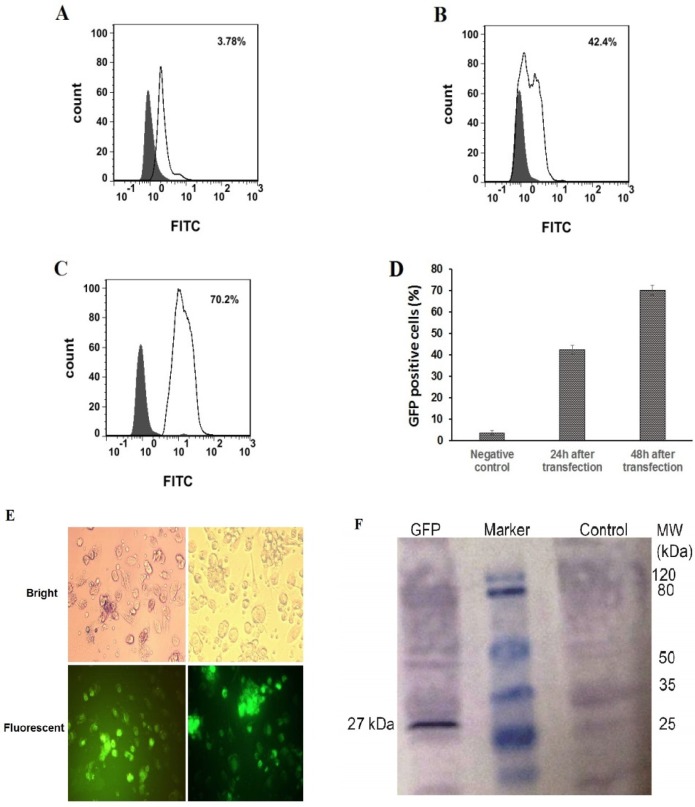
Flow cytometry analysis, fluorescence microscopic images and western blotting of GFP PLGA/PEI NPs-treated moDC cells. GFP PLGA/PEI nanoparticles were added to the cell culture media at the final concentration of 5 μg/mL and GFP protein expression was measured 24-48 h post treatment. (A-D) flow cytometry analysis of GFP PLGA/PEI NPs-treated moDCs. (A) As a control, moDCs only treated with PBS buffer (Negative Control). (B and C). Percentage of GFP positive moDCs were transfected with PLGA/PEI NPs encapsulation of GFP mRNA 24 h and 48 h after transfection respectively. The percentage number in the fluorescence- activated cell sorting profile represents the percentage of GFP-expressing cells sorted within a prefixed gate region. (D) The comparison percentage of GFP positive moDCs in control negative and after transfection of moDCs with PLGA/PEI NPs encapsulation of GFP mRNA. The results represent the mean ± SD (n = 3 for one of three independent experiments). *P *< 0.05 by One-way analysis of variance as compared with the corresponding controls. (E) GFP expression in moDCs using fluorescence microscopy. Immature moDCs were transfected with PLGA/PEI NPs encapsulation of GFP mRNA and were analyzed for GFP protein expression 48 h after transfection. GFP protein expressed in moDCs have been indicated in fluorescent microscopy fields (Down). (F) Western blotting for detect expression of GFP protein in moDCs 48 h after transfection by PLGA/PEI NPs encapsulation of GFP mRNA. The right line of marker is protein extract from the un-transfected DCs as negative control and the left line of marker is GFP protein with the expected molecular mass (27 kDa) in the DCs extract

To generate iDCs from monocytes, CD14+ cells were purified from normal blood samples and differentiated into iDCs using a human recombinant IL-4 and GM-CSF. On day 5, monocyte-derived cells were assessed for the expression of monocyte- and iDC-specific markers by flow cytometry. Supplementary1 indicates the result of a representative experiment. After 24 and 48 h *in-vitro* transfection of the immature ‎moDCs with re-suspended NPs in RPMI solution at a concentration of 5 μg/mL, ‎the expression of GFP in the moDCs was investigated. Our results demonstrate that GFP expression can be detected, using flow cytometry, fluorescent microscopy examination, and western blotting assay. The average percentage number in the fluorescence-activated cell sorting profile represents the percentage of the cells expressing GFP 24 and 48 h after transfection that was 42.4% ± 1.98, 70.2% ± 232, respectively (Figures 8A-8D). Microscopic examination revealed ‎that transfection of moDCs with PLGA/PEI NPs happened in a relatively good transfection ‎efficiency (Figure 8E). Also, the GFP expression in moDCs was screened by western blotting after ‎‎72 h (Figure 8F). 

As shown in Figure 8F, GFP protein with the expected molecular weight (27 kDa) was ‎observed in cell extracts obtained after transfection of moDCs. The right side of the marker shows ‎the expression of proteins in negative control cells‎ which were moDCs that did not exposed to mRNA-encapsulated NPs.

*Evaluation of PLGA/PEI NPs cytotoxicity*

**Figure 9:**
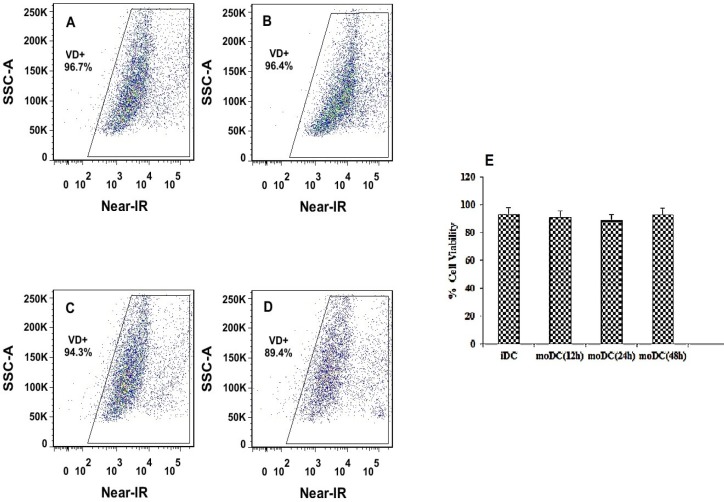
Flow cytometry analysis of moDCs viability, before and after transfection with NPs. The dot plots show Near-IR fluorescence on the x-axis and count cells on the y-axis. Gates were drawn based on un-staining Mo-DCs (row cells). Percentages of dead cells (left corner) and viable cells (right corner) are indicated. (A) The percent viability of iDC calls before transfection with PLGA/PEI nanoparticles. (B-D) The percent viability of moDC calls after transfection with PLGA/PEI nanoparticles at 12, 24 and 48 h after exposure respectively. (E) Comparison of the mean viability of moDCs cells before and after exposure to nanoparticles (at 12, 24, 48 h, respectively). Measurements were repeated in triplicate for each time and the standard errors are shown

The viability of labeled iDCs with Near-IR (viability dye) before and after transfection with PLGA/PEI NPs (at 12, 24 and 48 h after exposure) were estimated using a FACSAria II flow cytometer. Examination of mean fluorescent intensity of the cultured moDCS that were exposed to mRNA-encapsulated NPs showed a similar trend to the percentage of iDCs that were not exposed to mRNA-encapsulated NPs (Figures 9A-9D). The mean viability of the pre-treated moDCs (93.23 ± 1.64) and the treated moDCs (at 12, 24, 48 h: 91.25 ± 3.18, 88.45 ± 3.3, 92.83 ± 1.48 respectively), did not show any significant difference (Figure 9E).

**Table 1:** Size, Zeta potential, PDI, mRNA encapsulation efficiency (EE) and loading capacity (LC) of mRNA-loaded nanoparticle

**Formulation**	**Size (nm)**	**PDI** **a**	**Zeta Potential (mV)**	**EE (%)** **b**	**LC (%)** **c**
Blank PLGA/PEI NPs	428 ± 12	0.437 ± 0.012	12.9 ± 0.275	-	-
mRNA encapsulated PLGA/PEI NPs	606 ± 9.7	0.454 ± 0.084	12.2 ± 0372	73.54 ± 2.12	11.47 ± 0.33

aPolydispersity Index. bEncapsulation Efficiency. cLoading Capacity.

## Discussion

In recent years, mRNA has been proposed as an effective alternative to DNA-based treatments and successfully was used in vaccine technology due to several benefits. mRNAs do not need to enter nuclear to perform transfection process. The integration probability of mRNA into the host genome is low and also they have high expression rate and predicted kinetics. However, due to some undesirable features such as large size, negative charge, and enzymatic degradation, the widespread use of mRNA is limited. Therefore, it is possible to overcome these limitations by modifying mRNA delivery methods and use it as a therapeutic tool (5, 21). To formulate mRNA vaccines, many complex agents have been used to facilitate the delivery of mRNA to the cytosol. But the use of complex agents *‎in-vivo* is often associated with toxicity, especially for high molecular weight compounds (22). Current approaches to encapsulate and transfer of the therapeutic compounds focus on the development of liposomal and biodegradable polymeric nanoparticles (23, 24). In this regard, cationic lipids are commonly used, for example, to the intravenous injection and intradermal administration of antigen coding mRNA (25). The first report presented in order to increase the protection and uptake of mRNA by cells is ‎related to generation of an immune response by direct administration of mRNA encapsulated in liposomes. In this study, the mice were immunized intraperitoneally with liposomes containing mRNA, which encoded the influenza virus nucleoprotein (NP). The results of their study showed that injection of only mRNA or mRNA encapsulated in liposomes could not raise cytotoxic T lymphocytes (CTL) and suggested that the cause of the absence of CTL is due to the instability of liposomes in the peritoneal environment (26). The most important defect in the liposomal system is the short half-life of the nanoparticles in the serum for a few hours due to the binding of serum proteins to them. Therefore, in order to prevent instability caused by the interaction of cationic liposome with serum proteins in studies conducted on miRNA delivery, sometimes anionic and neutral liposomes were used. Also, in other studies, the linkage of the lipids with hydrophilic and flexible compounds such as polyethylene glycol (PEG) is also considered as a solution for increasing the stability and half-life of lipid nanoparticles (27). Although cationic lipid-based delivery systems are attractive in terms of performance, they might generate toxicity and induce immunogenicity *in-vitro* and *in-vivo* (1). Another report also showed that the complexity of mRNA with protamine was the most effective way of stabilizing mRNA against degradation with serum components (28). Protamine as a polycationic heterogeneous peptide is obtained from fish. Combining protamine with polyanionic heparin through electrostatic interaction neutralized the anticoagulant heparin functions (29). But there is evidence that the use of protamine is associated with a number of side effects, including complement activation, release of histamine, carboxypeptidase inhibition, production of thromboxane, nitric oxide, and antibody (30). The use of cationic polymers for delivery of synthetic mRNA has not been studied in ‎comparison ‎with pDNA and siRNA, but they have high potential and have been well developed to compete with many lipid systems. The negatively charged synthetic mRNA complexation spontaneously interact with cationic polymers due to their opposite charges, and typically polyplexes are more stable than lipoplexes. Cationic polymers are able to ‎bind and also ‎condense synthetic mRNA into NPs which makes IVT-mRNA uptake more efficient ‎through ‎endocytosis. Moreover, the nanostructures protect the synthetic mRNA from nuclease degradation, and facilitate ‎endosomal ‎escape (31). Among various cationic polymers, PLGA has been largely evaluated for its ability to deliver various types of drugs (32, 33). PLGA-composed nanoparticles easily escape from lysosomes to the cytoplasmic portion and release their content over a long period of time (34, 35). These features make PLGA nanoparticles effective as a potential tool for gene delivery (36, 37). PLGA-based nanoparticles were used as a carrier of miRNA for HepG2 cell transfection (38). Also, PLGA nanoparticles have been used as a delivery agent for vaccines. Many studies have shown that nanosystems can increase the absorption of antigens or adjuvants by APCs and provide better immune responses than soluble antigen presentations. Apart from higher absorption by APCs, and in particular the use of PLGA nanoparticles for vaccines and cancer immunotherapy based on nanotechnology, there are several other advantages (14). The advantage of this polymer is the ability to escape from the endosomes, its biodegradability, and cellular compatibility (39). PEI is one of the most efficient positively charged cationic polymers used as a gene carrier that condenses a nucleic acid into cationic polyplexes, and can stabilize and protect nucleic acid for hours from nucleases in the tissues (40).

PEI is only partly protonated in the extracellular neutral pH, so the nucleic acid is still linked ‎through electrolytic interactions, but in the acidic environment of the endosome, the protonation ‎and charge density of PEI would increase. This process will lead to the instability of the vesicles ‎containing the polyplex and then facilitate the release of the nucleic acid molecule from the ‎endosome, as a result, PEI provides a favorable component in the field of delivery of nucleic acids (41, 42). So far, nanoparticles containing PLGA and PEI polymers have been used in many studies to deliver the gene and drug. Patil and colleagues argued that PEI, as an amine-rich cationic polymer, has the potential to increase the SiRNA retention rate in the matrix of the PLGA. Also, the release of oligonucleotide from a PLGA/PEI nanoparticle compared with the PLGA nanoparticle, is more continuous. Polymer PEI is more hydrophilic than PLGA and is easily soluble in aqueous buffers, which creates channels in the nanoparticle matrix, accelerating the degradation of the nanoparticle and release of oligonucleotides. Their results indicated that insertion of PEI in PLGA nanoparticle affects the cell′s up-take of the nanoparticle. The effective efficiency of SiRNA-loaded nanoparticles in reducing the gene expression in the two cell lines suggests that the PLGA/PEI nanoparticles were a useful system for delivery of SiRNA. The problem with using PEI is its toxicity. PEI, depending on the concentration used, can be very toxic to the cells. The formulation used in above study had a low concentration of PEI (3 ng/µg of PLGA) and PLGA/PEI nanoparticles did not induce a significant cytotoxicity (43). We also used a similar concentration (1 ng/0.3 µg of PLGA) in the present study in nanoparticle formulation. In another study, Shengpeng Wang *et al.* used PEI-PLGA nanoparticles with hyaluronic acid to deliver doxorubicin (DOX) and miR-542-3p to breast cancer. Their results demonstrated that HA/PEI-PLGA nanoparticles were able to provide chemotherapeutic agents and miRNAs as tumor suppressor (44).

In this study, we implemented a simple, efficient, and controlled system using common strategies. The NPs we made are consisted of two bio-materials: PLGA and PEI. PLGA is a hydrophobic and biodegradable component with the ability to encapsulate many hydrophobic biomaterials such as nucleic acids. PEI improves the effectiveness of the encapsulation and promotes the interaction between positively charged nanoparticles and negatively charged cell membranes and also it elevates the release of encapsulated biomolecules and endosomal escape. The branched PEI with higher molecular weights has shown great success for *in-vitro* transfections since it condenses nucleic acids more efficiently than the linear PEI, hence to design our NP we used branched PEI (45). Here we showed that PLGA encapsulated mRNA/PEI complex, had no toxic effect on immature DCs and was capable of delivering IVT-mRNA in DC cells. PLGA/PEI nanoparticles may further be studied as a vehicle for the delivery of various synthetic mRNA to DC in order to provide a variety of recombinant proteins to stimulate the immune system. Particle size and zeta-potential are significant factors since they directly have effect on the stability, biodistribution and also these factors determine the level of cellular and tissue uptake.

Zeta potential is an indicator of the surface charge of NPs/IVT-mRNA complexes. The surface positive charges of the nanostructures can aid the NPs attach firmly to the negatively charged cellular membrane, hence accelerating their entry into the cells through endocytosis. As presented in Table 1, PEI is located on the surface of PLGA NPs owing to the electrostatic interaction of anionic PLGA copolymers with cationic PEI molecules. Introduction of PEI resulted in the positive zeta potential of the NPs. IVT-mRNA are attached to the surface of PLGA/PEI NPs because of the electrostatic interaction between positively charged PLGA/PEI NPs and negatively charged IVT-mRNA. The zeta potential of the complexes enhances in parallel with the NPs/ IVT-mRNA N/P ratio, which leads a reasonable affinity to the surface of the cells.

## Conclusion

We developed PLGA/PEI NPs which contain a hydrophobic PLGA core and PEI as a cationic polymer. The NP formulations were characterized and evaluated for delivery of IVT-mRNA. NPs were well tolerated by human monocyte- derived DCs (moDCs). The nanoparticle was prepared by a simple and controllable process and contained biocompatible, biodegradable, and non-toxic polymers and hence might be useful as a potential IVT-mRNA delivery system. Further, *in-vivo* investigations are planned to show the efficacy of these nanostructures as an IVT-mRNA delivery system for a variety of applications, especially for the presentation and delivery of recombinant proteins by IVT-mRNA to dendritic cells with therapeutic aims.
